# Faster, More Reproducible DESI-MS for Biological Tissue Imaging

**DOI:** 10.1007/s13361-017-1714-z

**Published:** 2017-06-15

**Authors:** Jocelyn Tillner, Vincen Wu, Emrys A. Jones, Steven D. Pringle, Tamas Karancsi, Andreas Dannhorn, Kirill Veselkov, James S. McKenzie, Zoltan Takats

**Affiliations:** 1 Computational and Systems Medicine, Department of Surgery and Cancer, Faculty of Medicine, Imperial College London, Sir Alexander Fleming Building, South Kensington, London, SW7 2AZ UK; 2 NiCE-MSI, National Physical Laboratory, Hampton Road, Teddington, TW11 0LW UK; 3 Waters Corporation, Altrincham Road, Wilmslow, SK9 4AX UK; 4 Waters Research Center, Záhony utca 7., C ép., 1. em., 1031 Budapest, Hungary

**Keywords:** DESI-MS, Mass spectrometry imaging, Fast DESI, Desorption electrospray ionization

## Abstract

A new, more robust sprayer for desorption electrospray ionization (DESI) mass spectrometry imaging is presented. The main source of variability in DESI is thought to be the uncontrolled variability of various geometric parameters of the sprayer, primarily the position of the solvent capillary, or more specifically, its positioning within the gas capillary or nozzle. If the solvent capillary is off-center, the sprayer becomes asymmetrical, making the geometry difficult to control and compromising reproducibility. If the stiffness, tip quality, and positioning of the capillary are improved, sprayer reproducibility can be improved by an order of magnitude. The quality of the improved sprayer and its potential for high spatial resolution imaging are demonstrated on human colorectal tissue samples by acquisition of images at pixel sizes of 100, 50, and 20 μm, which corresponds to a lateral resolution of 40–60 μm, similar to the best values published in the literature. The high sensitivity of the sprayer also allows combination with a fast scanning quadrupole time-of-flight mass spectrometer. This provides up to 30 times faster DESI acquisition, reducing the overall acquisition time for a 10 mm × 10 mm rat brain sample to approximately 1 h. Although some spectral information is lost with increasing analysis speed, the resulting data can still be used to classify tissue types on the basis of a previously constructed model. This is particularly interesting for clinical applications, where fast, reliable diagnosis is required.

**Electronic supplementary material:**

The online version of this article (doi:10.1007/s13361-017-1714-z) contains supplementary material, which is available to authorized users.

**Graphical Abstract:**
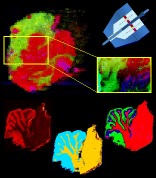
ᅟ

## Introduction

Since its first publication in 2004 [1], desorption electrospray ionization (DESI) mass spectrometry (MS) has seen a large rise in popularity and extension into a wide field of applications [2]. One such application is the MS imaging of biological tissue specimens, it being particularly well suited for the spatial mapping of lipids (fatty acids, phospholipids, and diglycerides and triglycerides) [3]. By acquisition of the MS profile of different tissue types, these tissues can be characterized and, with use of either characteristic compounds or multivariate statistical tools, classified [4–9]. This makes DESI a promising method for molecular pathology applications, whereby tissue types are characterized by their biochemical composition as opposed to morphological features as in classic histopathology. To ensure the accuracy of these methods, high quality and repeatability are desirable for DESI data. Two recent studies have looked at the repeatability and reproducibility of DESI-MS. In the first, a Versailles Project on Advanced Materials and Standards (VAMAS) interlaboratory study performed by the National Physical Laboratory [10], the absolute intensity repeatability was measured with glass slides coated with rhodamine B and with double-sided adhesive tape. The spectral shape, given by the relative intensity of peaks in selected regions, was compared with use of the tape. Absolute intensity repeatability varied largely between the participating laboratories from 14% to 140% (average 51%) for rhodamine B and from 14% to 58% for adhesive tape samples (average 31%). The spectral shape showed an average repeatability of 11%. Day-to-day reproducibility of the spectral shape was 31%. In the second study, by Abbassi-Ghadi et al. [11], the repeatability and reproducibility of DESI were assessed by the imaging of human esophageal cancer tissue sections. They were calculated from the variation in normalized intensities of 25 lipid species in the *m*/*z* range from 600 to 900. Repeatability was calculated as 22% and reproducibility was calculated as 20%.

**Figure 1:**
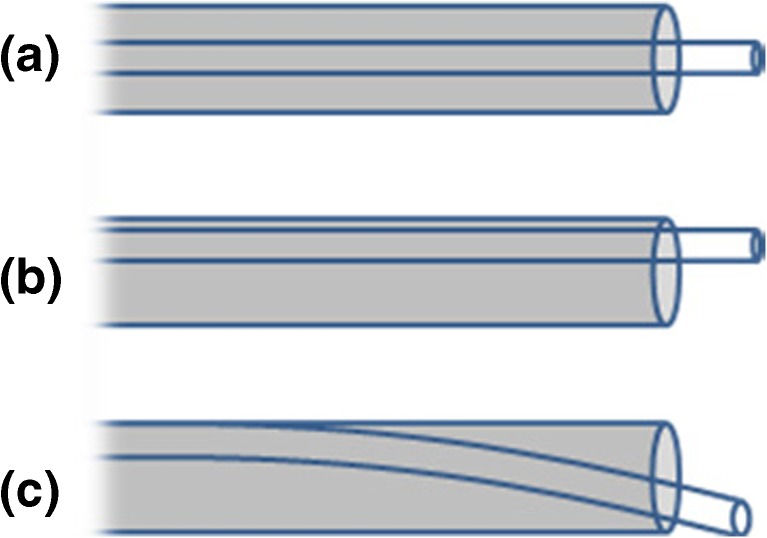
Positioning of the solvent capillary in the laboratory-built desorption electrospray ionization (DESI) sprayer. Ideally, the fused-silica solvent capillary should be concentric with the gas capillary (*a*), resulting in rotational symmetry and an even distribution of the nebulization gas flow. Because of the flexibility of the fused silica, however, it is usually off-center (*b*) or bent and protruding at an angle (*c*)

One major source of variability in DESI-MS is thought to be the geometry of the sprayer, which produces the primary electrospray [12]. Although a number of studies have examined the optimization of solvent composition [13, 14] and flow rate and geometrical parameters [15–17] for improved spatial resolution and DESI performance, the design of the sprayer itself has remained largely unchanged. There are two commonly used DESI sprayer designs: Firstly, the original design, initially published for electrosonic spray ionization [18], consisting of two ideally concentric fused silica capillaries. This is the design predominantly used for tissue imaging applications. Secondly, the commercially available design from Prosolia, which consists of a stainless steel gas nozzle with a fused-silica solvent capillary. Both designs use a straight-edged fused-silica capillary as the solvent capillary, which is fixed by a ferrule several centimeters away from the spray tip. The main disadvantage of the fused-silica capillary is its flexibility. Rather than being concentric with the gas capillary or gas nozzle aperture, the solvent capillary usually leans to one side (Figure 1, schematic b). It can also bend, causing it to protrude at an angle (Figure 1, schematic c). This causes a loss of rotational symmetry, introducing new, uncontrollable degrees of freedom to the setup, and creates an asymmetrical, unevenly distributed nebulization gas flow. Consequently, the size distribution of the primary electrospray droplets is enlarged [19], which can in turn result in a reduction in ionization efficiency. At certain gas flows, turbulence can also cause the solvent delivery capillary to vibrate. This type of vibration can be exploited for droplet generation at low solvent flow rates, such as in the oscillating capillary nebulizer [20], but is undesirable in DESI. Pasilis et al. [21]observed a dependence of signal intensity on the scanning direction, and suggested that this may be due to asymmetry of the primary electrospray. This was recently confirmed in a study with a sprayer in which the solvent capillary was fixed to the side of the gas capillary [12]. It has been suggested in several instances that the ideal DESI sprayer would probably allow perfect centering of the solvent capillary within the gas capillary [12, 21, 22]. However, in practice this can be difficult to achieve.

In addition to geometrical issues, laboratory-built sprayer capillaries are usually cut by hand, which means that the edge can contain small imperfections that can have an impact on the quality of the electrospray. A stainless steel solvent capillary could also be used; however, this can lead to capacitance effects (charging and discharging to the gas nozzle), which can affect ionization. It can also lead to corona discharge and arcing between the sprayer and other metal components of the DESI source.

Here, we examine redesigning the DESI sprayer to provide better stability of solvent capillary positioning and thus better reproducibility. This includes the use of a TaperTip™ emitter. Tapered fused-silica emitters were used previously for DESI by Van Berkel et al. [23] in 2005 to improve the spatial resolution of DESI. However, without accurate positioning, the reproducibility of the primary electrospray is limited. In an initial experiment, the fused-silica solvent capillary of the laboratory-built sprayer was replaced to increase its wall thickness and, thereby, it stiffness. The use of machine-cut TaperTip™ emitters was also examined both for reproducibility and for improved spatial resolution. The results of these experiments led to a new sprayer design with a 20-μm inner diameter (ID) tapered fused-silica emitter to allow a stable primary electrospray at very low flow rates, and comprising a device to improve positioning accuracy of the emitter and reproducibility.

## Experimental

### Solvents and Samples

Methanol and water were liquid chromatography–MS grade and were purchased from Sigma-Aldrich (St Louis, MO, USA). Rhodamine B coated slides were kindly provided by the National Physical Laboratory (Teddington, UK), and were prepared as in the recently published VAMAS interlaboratory study [10]. Pork liver samples were obtained from a local supermarket. Rat cerebellum was from a control animal used in a study by collaborators at AstraZeneca. Details can be found in Swales et al. [24]. The colorectal cancer samples were analyzed within the scope of a larger clinical study, which received full ethics approval (14/EE/0024). All tissue samples were fresh frozen on dry ice and stored at -80 °C until processing. For DESI-MS, samples were cryosectioned at 10-μm thickness and thaw-mounted onto SuperFrost Plus microscope slides (Thermo Fisher Scientific, Waltham, MA, USA). Pork liver was used for optimization of DESI parameters. Three adjacent colorectal cancer tissue sections were used to test different pixel sizes. Five adjacent rat cerebellum sections were used to assess high MS scan speeds and DESI imaging velocities. All sections were subsequently submitted to the Research Histology Facility at the Imperial College South Kensington Campus for hematoxylin and eosin (H&E) staining and subsequent histological examination.

### Sprayers

The traditional laboratory-built sprayers were built from a 1/16-in. stainless steel tee (Swagelok, Solon, OH, USA) with a fused-silica gas capillary [363-μm outer diameter (OD), 220-μm ID], and a fused-silica solvent capillary (150-μm OD, 50-μm ID; both were obtained from SGE Analytical Science, Milton Keynes, UK), held in place with NanoTight™ sleeves (Idex, Lake Forest, IL, USA). To assess the impact of solvent capillary stiffness, altered sprayers with a fused-silica gas capillary of 700-μm OD and 530-μm ID and a fused-silica solvent capillary of 360-μm OD and 50-μm ID (both obtained from SGE Analytical Science, Milton Keynes, UK) were built. A further variant, where the solvent capillary was replaced with a TaperTip™ emitter (360-μm OD, 50-μm ID, from New Objective, Woburn, MA, USA) was also tested. A schematic for these sprayers can be found in Fig. S1.

**Figure 2:**
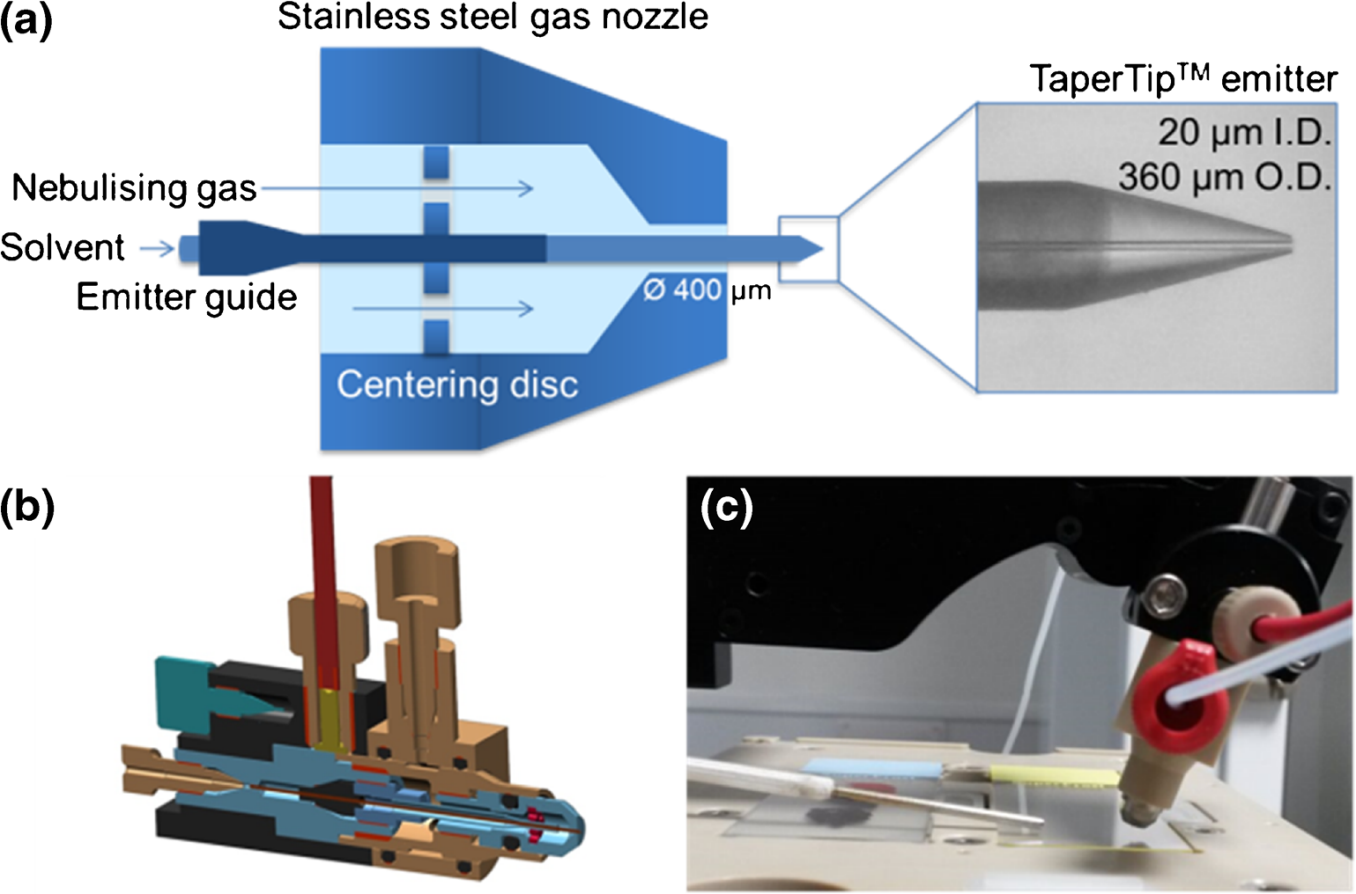
Geometrical configuration of the novel DESI sprayer. **a** The emitter, gas nozzle, and emitter guide. The TaperTip**™** emitter is held in a stainless steel emitter guide, which slides into a positioning disc attached to the stainless steel gas nozzle. This positions the emitter, while at the same time allowing the nebulizing gas to flow through radially arranged holes. **b** CAD drawing of the novel sprayer design. **c** The DESI setup with the novel sprayer on the Xevo G2-XS QTof instrument. *I.D*. - inner diameter, *O.D*. - outer diameter

The commercial sprayer was obtained from Prosolia (Indianapolis, IN, USA) and used as provided. The novel DESI sprayer was developed and manufactured in collaboration with Waters (Milford, MA, USA). It consisted of a stainless steel gas nozzle with an aperture of 400 μm and a TaperTip™ emitter (New Objective, Woburn, MA, USA) with an OD of 360 μm and an ID of 20 μm. This emitter is designed to deliver a stable electrospray at flow rates from 0.2 to 3 μl/min. The emitter was secured by a swaged stainless steel emitter guide and held with a stainless steel positioning disc with radially arranged holes for the nebulizing gas flow (Figure 2). This disc was coated with an electrically insulating material to prevent leakage currents flowing and charging of the stainless steel gas nozzle. Schematic drawings of the sprayer construction can be found in Fig. S2.

### DESI-MS Instrumentation

All experiments were performed with a Xevo G2-XS QTof instrument (Waters, Milford, MA, USA) operated in sensitivity mode and equipped with a two-dimensional DESI stage from Prosolia (Indianapolis, IN, USA) and a custom-built inlet capillary heated to 490 °C. DESI parameters were optimized for the best signal intensity on tissue and were as follows: spray voltage, 4.5 kV; solvent, 95:5 methanol–water; flow rate, 1.5 or 0.5 μl/min for pixel sizes of 100 μm or 50 and 20 μm respectively; nebulizing gas, nitrogen; gas pressure, 7 and 4.5 bar for 1.5 and 0.5 μl/min flow rate respectively; sprayer incidence angle, 75°; collection angle, 10°; sprayer-to-inlet distance, 10 mm; sprayer-to-sample distance, 1.5 mm. MS parameters were as follows: scan time (unless specified otherwise), 1 s; source temperature, 120 °C; sampling cone voltage, -40 V; source offset, -80 V.

### Data Acquisition

For the comparison of spectral pattern repeatability, five traditional laboratory-built sprayers, six altered laboratory-built sprayers (three with blunt capillaries, three with TaperTip™ capillaries), three commercially available (Prosolia) sprayers, and three novel sprayers were compared with use of adjacent sections from the same pork liver sample analyzed in randomized order in negative ion mode. Three sections were analyzed with each sprayer. Five lines were acquired across each section at a velocity of 100 μm/s and with a line-to-line spacing of 500 μm to avoid overlap. All tissue imaging data acquired with the novel sprayer were acquired with HDImaging version 1.3.5 in combination with MassLynx version 4.1 (Waters, Milford, MA, USA). The colorectal sample was acquired at a scan speed of one scan per second, and the stage speed was adapted accordingly to achieve pixel sizes of 100, 50, and 20 μm. For the rat cerebellum samples, the scan speed was increased from one scan per second to 5, 10, 20, and 30 scans per second. The stage scan speed was adapted accordingly to maintain a constant pixel size of 50 μm.

### Data Processing

Line scan raw files were converted into mzML files with the ProteoWizard msConvertGUI (Vanderbilt University, Nashville, TN, USA), and then into an imzML image file with imzML Converter version 1.3 [25]. The imzML files were imported into a MATLAB environment for subsequent analysis. The spectra for each line of a tissue section were averaged. Spectral pattern reproducibility was calculated according to the method of Dill et al. [26]. Briefly, the overall mean spectrum is calculated and all spectra are converted into *n*-dimensional vectors (with *n* being the number of spectral features) and normalized to a unit length. Each spectrum is then multiplied by the mean spectrum to obtain a similarity score. The relative standard deviation (RSD) of this score corresponds to the spectral pattern reproducibility.

Imaging data were analyzed directly in HDImaging or concatenated raw files were centroided and lock mass corrected with MassLynx version 4.1. They were imported into MATLAB, co-registered with the optical image of the H&E-stained section [4], and annotated by a histopathologist. For principal component analysis, the data were normalized by median fold change normalization and log transformed for variance stabilization (for details see [27]). We performed *k*-means clustering on nonnormalized, nontransformed data in two steps: first, 2-means clustering was used to separate the tissue from the background. Then *k*-means clustering was performed again on the tissue area. Clustering was performed with a squared Euclidean distance measure. The number of spectral features for each image was measured by our picking 25 pixels each for white matter and gray matter in roughly the same region in all images and extracting the spectral information for those pixels from the unprocessed raw data.

### Tissue Prediction

The tissue prediction potential of the high scan rate data was evaluated. With use of the histopathologist’s annotations for the one scan per second tissue section, annotated pixels were normalized to the total ion count (TIC) and used as the training set in a leave-one-class-out recursive maximum margin criterion (MMC) discriminatory model [28]. For all but the one scan per second tissue section, peak alignment was performed to match spectral variables across the five tissue sections. The *m*/*z* vector from each section was mapped to the *m*/*z* vector of the one scan per second section with a maximum permitted *m*/*z* shift of 50 ppm. All pixels were TIC normalized and predicted against the MMC linear discriminant analysis model.

For each of the four annotated classes (white matter, gray matter 1, gray matter 2, background) a classification probability between 0 and 1 was derived for each pixel. Pixels with a single high probability (e.g., greater than 0.8) can be confidently assigned, those with multiple high probabilities can be ambiguously classified (e.g., at a tissue intersection), and those with multiple low probabilities cannot be classified.

## Results and Discussion

Spectral pattern reproducibility of the conventional laboratory-built sprayer and the commercially available sprayer (Prosolia) was assessed according to the method of Dill et al. [26] (see Table 1). Performance varied greatly between laboratory-built sprayers from 0.39% to 1.81% RSD, and spectra were not consistent between sprayers (RSD of 5.06%). The commercial sprayers from Prosolia showed worse intrasprayer repeatability (RSD of 0.49–2.43%), but better intersprayer repeatability (RSD of 3.86%). A lot of the variability was thought to be attributable to variability in solvent capillary positioning. To test this, altered laboratory-built sprayers with thick-walled solvent capillaries (wall thickness 335 μm, compared with 50 μm in conventional sprayers) were built and tested for spectral pattern repeatability. Both for individual sprayers and across sprayers, repeatability was an order of magnitude higher than with the traditional design. Sensitivity was also increased about twofold. For comparison, TaperTip™ emitters, which have the advantage of having a machine-cut, tapered tip, were also tested. Repeatability was not found to be significantly better, and across sprayers it was slightly lower than for the blunt-tipped sprayers. However, this could be because the TaperTip™ emitters produced a smaller spray point and thus sample a smaller tissue area, which would make them more prone to variability caused by tissue inhomogeneity.

**Table 1:** Spectral pattern repeatability (relative standard deviations) for pork liver in negative ion mode for different sprayer designs

	Spectral pattern repeatability (%)	
	Within sprayer	Across sprayers
Conventional laboratory-built sprayer (*n* = 5)	0.39–1.81 (average 0.82)	5.06
Prosolia sprayer (*n* = 3)	0.49–2.43 (average 1.28)	3.86
Altered laboratory-built sprayer (blunt capillary) (*n* = 3)	0.02–0.08 (average 0.05)	0.18
Altered laboratory-built sprayer (TaperTip™ emitter) (*n* = 3)	0.02–0.16 (average 0.06)	0.38
Novel sprayers (*n* =3)	0.05–0.25 (average 0.14)	0.40

Repeatability was improved by an order of magnitude by our increasing the stiffness of the solvent capillary and reproducible positioning, thus reducing geometrical variability. A schematic for the laboratory-built sprayers can be found in Fig. S1. A detailed drawing of the novel sprayer can be found in Fig. S2

The results from these experiments were then used to design a new sprayer. The fused-silica gas capillary was replaced with a metal cone for robustness. A 20-μm-ID (360-μm-OD) TaperTip™ emitter was used, as this provides good stiffness, a machine-cut tip, and flow stability at low flow rates, which is required to improve spatial resolution. The aperture in the gas cone was set to 400 μm, as a small aperture was found to improve the focusing of the primary electrospray (and thus, potentially, spatial resolution). A capillary positioning device, composed of a stainless steel emitter guide and a centering disc with concentric holes for the gas flow, was inserted (Figure 2) to fix the position of the emitter and to facilitate the insertion of the TaperTip™ through the gas cone aperture. The disc was coated with an insulating material to avoid electrical discharge and charging of the cone. Three of these novel sprayers were then also compared for spectral pattern repeatability. All three sprayers performed excellently, with a spectral pattern reproducibility of 0.25% or below per sprayer and 0.40% between them. Overall, the data demonstrate that fixing the emitter position using a stiffer capillary and an emitter guide leads to a more reliable geometry and thus increased spectral reproducibility.

**Figure 3:**
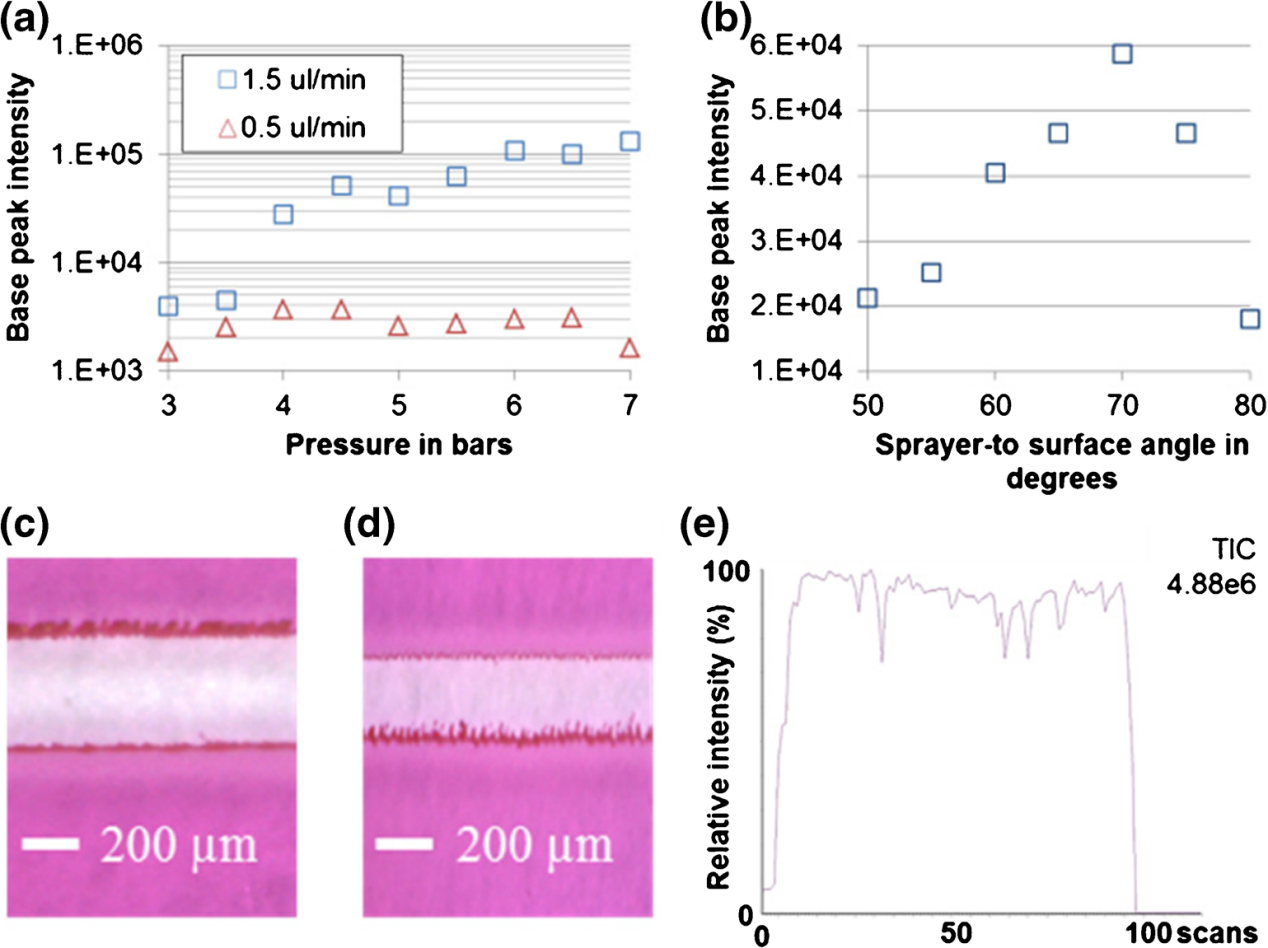
Optimization of gas pressures and spray angles with use of pork liver sections. **a**, **b** Base peak intensity for different nebulizing gas pressures at 1.5 μl/min and 0.5 μl/min (**a**) and different sprayer-to-surface angles at 1.5 μl/min and 7 bar (**b**), **c**, **d** Line scan desorption traces on a coating of rhodamine B at 1.5 μl/min and 7 bar and 0.5 μl/min and 4.5 bar at 100 μm/s and 50 μm/s respectively. **e** Example line scan for pork liver (1.5 μl/min, 7 bar, sprayer angle 70°). *TIC - Total Ion Count*

DESI-MS parameters (geometrical parameters, solvent flow rate, and gas pressure) were optimized for the novel sprayer before imaging. Pork liver sections were used. Parameters were adjusted for the highest lipid signal intensity (for the final parameter set, see “Experimental”). A nitrogen pressure of 7 bar was optimum for a solvent flow rate of 1.5 μl/min, while lower gas pressures in the range of 4–4.5 bar were optimum for a reduced flow rate of 0.5 μl/min (Figure 3a). Even with very little optimization, high-quality lipid spectra were obtained from the tissue with the novel sprayer. The optimal sprayer-to-surface angle was 70° (Figure 3b). The stability of the primary electrospray at different flow rates was assessed by line scanning across glass slides coated with rhodamine B and examination of the desorption trace (Figure 3c, d), and both the signal intensity and the signal stability were assessed by line scanning across pork liver at 100 μm/s (Figure 3e). The signal stability was satisfactory for both sets of operating conditions tested (1.5 μl/min, 7 bar and 0.5 μl, 4.5 bar), although signal intensity was reduced proportionally with the reduction of solvent flow rate. This is likely due to a reduction in the surface area being sampled.

**Figure 4:**
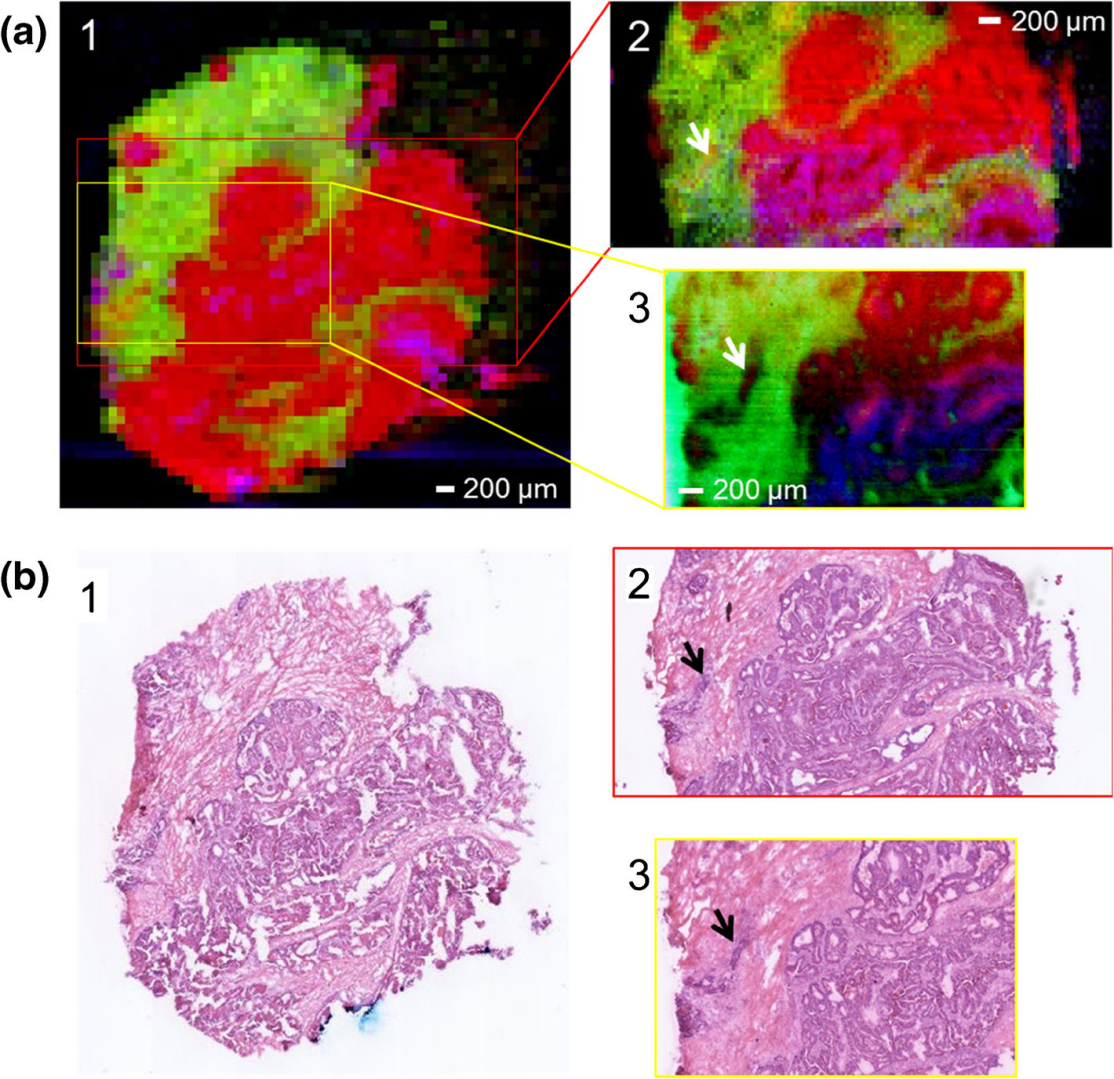
DESI mass spectrometry (MS) imaging of adjacent colorectal cancer sections at different spatial resolutions. **a** RGB images for the first three components of principal component analysis for images acquired at 100 μm (*1*), 50 μm (*2*), and 20 μm (*3*) pixel size. **b** Corresponding optical images of the same sections after hematoxylin and eosin (H&E) staining. The small tumor region marked with an *arrow* is approximately 50 μm × 250 μm in size

To assess the imaging performance of the novel sprayer, three images of adjacent colorectal samples were acquired. DESI stage velocities of 100, 50, and 20 μm/s (with one scan per second) were tested, resulting in images with pixel sizes of 100, 50, and 20 μm respectively. Solvent flow parameters were varied accordingly (see “Experimental”). Although signal intensity was reduced proportionally with the reduction in solvent flow rate, it was still sufficiently high to obtain high-quality images down to a pixel size of 20 μm (Figure 4). This allowed features as small as 40–60 μm (two to three pixels) to be resolved, which is similar to the highest resolution published in the literature [16].

**Figure 5:**
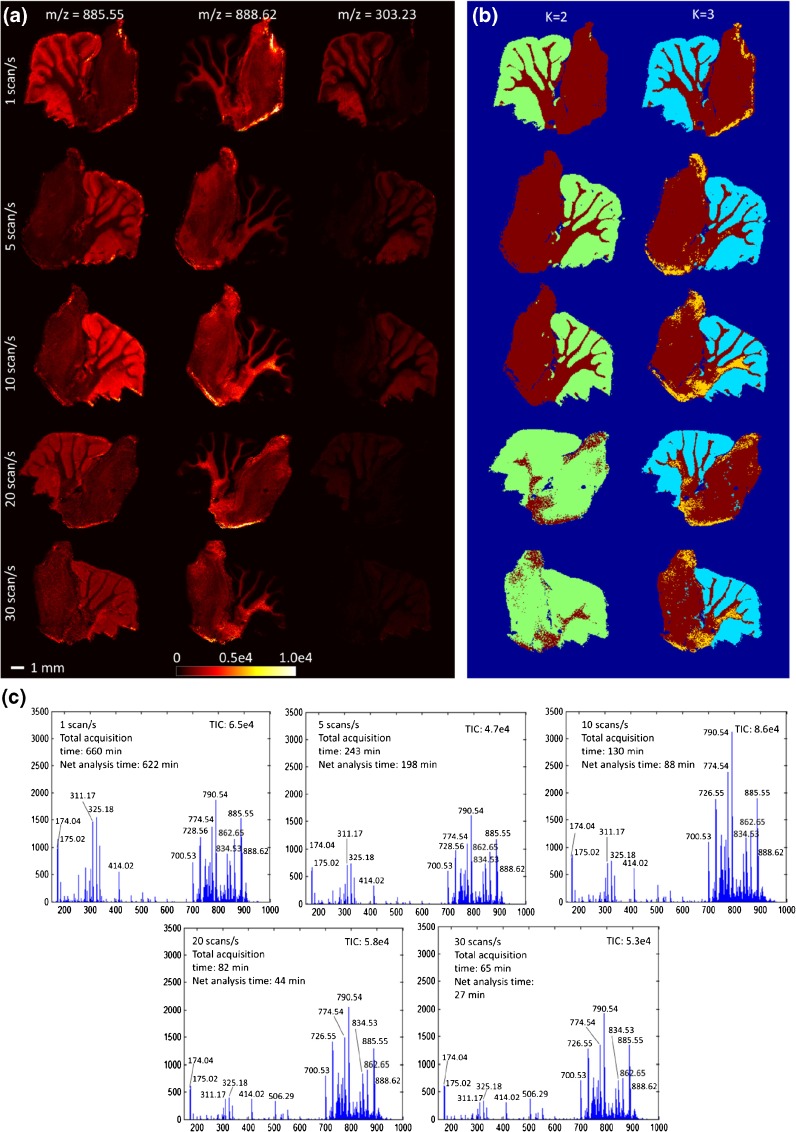
Fast DESI of rat cerebellum in negative ion mode at 50 μm × 50 μm pixel size. Selected ion images (**a**), *k*-means clustering images (**b**), and mean tissue spectra (scaled to the same intensity) (**c**) for DESI-MS analysis at five different scan speeds. Sections are consecutive but were not all mounted in the same orientation. Net analysis time is total acquisition time minus stage turnaround time

One major limitation of DESI, and MS imaging in general, is its long acquisition time. Conventionally, one to five pixels are acquired per second, resulting in a total analysis time of several hours for samples with diameters in 3–10 mm range. By using the novel sprayer in combination with a time-of-flight analyzer, we were able to shorten the acquisition time more than tenfold, the key contribution of the sprayer being its ability to provide a high ion yield from a small sampling spot size. To compare the impact of increased scanning speed on data quality, five adjacent sections from the same rat cerebellum, which were approximately 10 mm × 10 mm in size, were imaged at a nominal pixel size of 50 μm × 50 μm and at mass spectrometer scan speeds of 1, 5, 10, 20, and 30 scans per second. Selected ion images for *m*/*z* 885.55, 888.62, and 303.23 [assigned as phosphatidylinositol (18:0/20:4), sulfogalactosylceramide (42:2), and arachidonic acid] can be found in Figure 5, together with the mean tissue spectrum for each section and *k*-means clustering images. The TIC of the mean spectrum did not decrease significantly with an increase in scan speed, although the number of mass spectral features and the relative ratio of spectral features did change. Lower *m*/*z* ions, such as fatty acids, were affected by this change more strongly than higher *m*/*z* ions. This could be due to different levels of ion transmission efficiency both with increasing stage speed and with increasing mass spectrometer scan speed. Increasing the scan speed had some impact on mass resolution, as this is directly related to spectral intensity. Details on this can be found at the end of the electronic supplementary material.

Very similar results were obtained with 2-means clustering of the tissue area at scan speeds of 1, 5, and 10 scans per second, with a clear separation of white and gray matter. At higher scan speeds, this separation could be achieved only through introduction of a third cluster. This suggests that some spectral information is lost with an increase in scan speed. However, a separation of tissue areas is still easily achievable. Examination of the raw data shows that the overall number of spectral features decreases as the scan speed increases, although in some regions it then starts rising again (see Fig. S3). This rise is likely to be an artifact due to a decrease in signal intensity, which eventually leads to peak shape deterioration and peak splitting (see Fig. S4).

**Figure 6:**
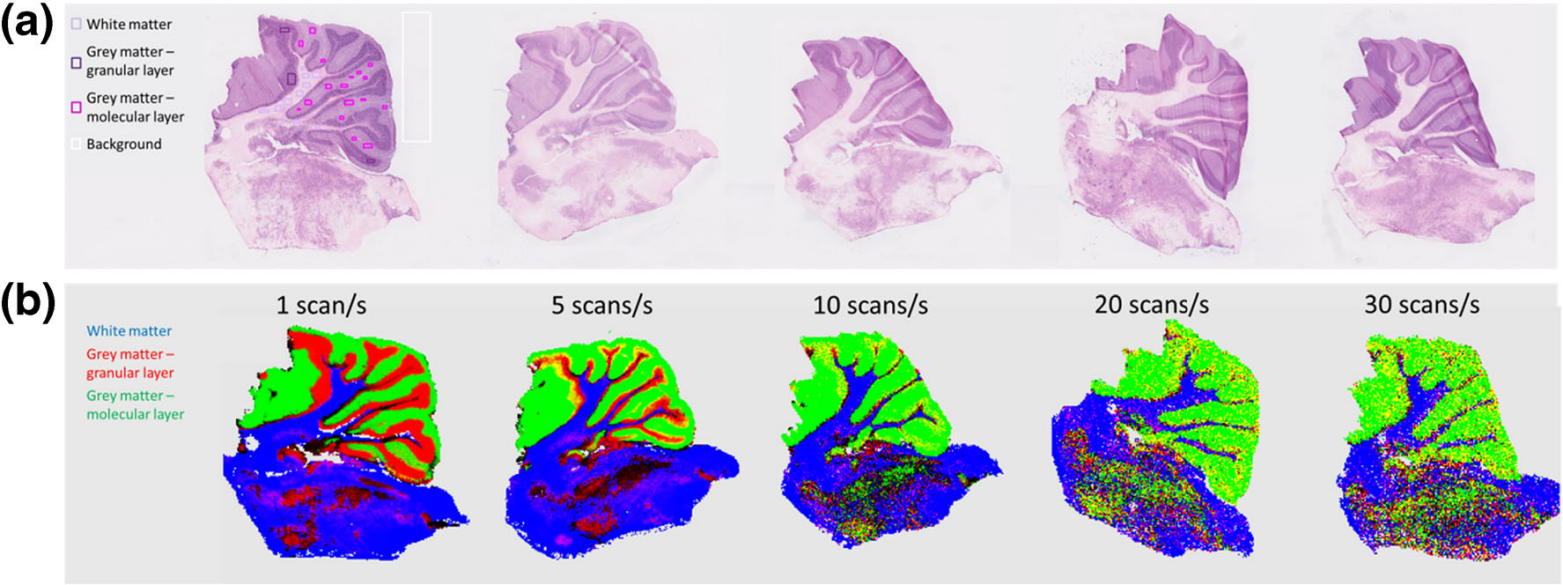
Maximum margin criterion (MMC) tissue prediction of rat cerebellum using fast DESI data. **a** Optical images of H&E-stained sections with histological annotations for the section analyzed at one scan per second. **b** MMC-predicted images for sections analyzed at different scan speeds. Unclassified pixels are *black*

To evaluate whether the high scan rate data could be used for accurate tissue prediction, the section analyzed at one scan per second was annotated on the basis of the optical image of the H&E-stained section. Annotated pixels were then used as a training set to predict the rest of the section and the four other sections with use of a one-against-all recursive MMC discriminatory model. Images with a continuous probability scale are displayed in Figure 6b. Images for fixed probability thresholds from 0.1 to 1 can be found in Fig. S5. The prediction of the same section shows excellent agreement with the optical image of the stained section. Prediction of the pixels deteriorated as scan speed increased. In particular, a separation of the two annotated types of gray matter was not possible above a scan rate of five scans per second. This is probably because the two types show related chemical profiles. However, classification accuracy was above 85% for white matter in all cases. Confusion matrices can be found in Fig. S6.

## Conclusion

The new sprayer presented here provides a viable alternative to the traditional laboratory-built design. It delivers high-quality lipid spectra and good stability over time with improved resolution. The use of a thick-walled, machined-tapered fused-silica emitter with an emitter guide to fix its position eliminates some of the major factors thought to contribute to DESI variability—namely, the surface qualities and the positioning of the emitter within the gas capillary or nozzle. The present sprayer provides high reproducibility, sensitivity, and spatial resolution, making possible fast and accurate DESI analysis. Acquisition of an image at a nominal pixel size of 20 μm allowed us to clearly resolve features as small as 50 μm across. Although some spectral information is lost at very high scan speed, different tissue areas can still be separated. Use of a high-quality training set allows rough tissue type prediction even in samples acquired at very high speed. This sprayer is therefore very promising for tissue analysis in a clinical context where a fast classification is needed. In practice, a database could be built from a reference sample set with classic scan parameters. Test samples could then be analyzed at high speed and classified with the database, allowing high-throughput diagnostics, which can be crucial in a surgical setting. In-depth analysis could then also be performed with traditional scan speeds.

## Electronic supplementary material

Below is the link to the electronic supplementary material.

ESM 1 (DOCX 2005 kb)

## References

[1] Takats Z, Wiseman JM, Gologan B, Cooks RG (2004). Mass spectrometry sampling under ambient conditions with desorption electrospray ionization. Science..

[2] Ifa DR, Wu CP, Ouyang Z, Cooks RG (2010). Desorption electrospray ionization and other ambient ionization methods: current progress and preview. Analyst..

[3] Eberlin LS, Ferreira CR, Dill AL, Ifa DR, Cooks RG (2011). Desorption electrospray ionization mass spectrometry for lipid characterization and biological tissue imaging. Biochim. Biophys. Acta..

[4] Veselkov KA, Mirnezami R, Strittmatter N, Goldin RD, Kinross J, Speller AVM, Abramov T, Jones EA, Darzi A, Holmes E, Nicholson JK, Takats Z (2014). Chemo-informatic strategy for imaging mass spectrometry-based hyperspectral profiling of lipid signatures in colorectal cancer. Proc. Natl. Acad. Sci. U. S. A..

[5] Eberlin LS, Norton I, Dill AL, Golby AJ, Ligon KL, Santagata S, Cooks RG, Agar NYR (2012). Classifying human brain tumors by lipid imaging with mass spectrometry. Cancer Res..

[6] Eberlin LS, Tibshirani RJ, Zhang J, Longacre TA, Berry GJ, Bingham DB, Norton JA, Zare RN, Poultsides GA (2014). Molecular assessment of surgical-resection margins of gastric cancer by mass-spectrometric imaging. Proc. Natl. Acad. Sci. U. S. A..

[7] Guenther S, Muirhead LJ, Speller AV, Golf O, Strittmatter N, Ramakrishnan R, Goldin RD, Jones E, Veselkov K, Nicholson J, Darzi A, Takats Z (2015). Spatially resolved metabolic phenotyping of breast cancer by desorption electrospray ionization mass spectrometry. Cancer Res..

[8] Abbassi-Ghadi N, Veselkov K, Kumar S, Huang J, Jones E, Strittmatter N, Kudo H, Goldin R, Takats Z, Hanna GB (2014). Discrimination of lymph node metastases using desorption electrospray ionisation-mass spectrometry imaging. Chem. Commun..

[9] Wiseman, J.M., Puolitaival, S.M., Takáts, Z., Cooks, R.G., Caprioli, R.M.: *Mass* Spectrometric profiling of intact biological tissue by using desorption electrospray ionization. Angew. Chem. **117**(43), 7256–7259 (2005) 10.1002/anie.200502362 16259018

[10] Gurdak E, Green FM, Rakowska PD, Seah MP, Salter TL, Gilmore IS (2014). VAMAS interlaboratory study for desorption electrospray ionization mass spectrometry (DESI MS) intensity repeatability and constancy. Anal. Chem..

[11] Abbassi-Ghadi N, Jones EA, Veselkov KA, Huang JZ, Kumar S, Strittmatter N, Golf O, Kudo H, Goldin RD, Hanna GB, Takats Z (2015). Repeatability and reproducibility of desorption electrospray ionization-mass spectrometry (DESI-MS) for the imaging analysis of human cancer tissue: a gateway for clinical applications. Anal. Methods..

[12] Tillner J, McKenzie JS, Jones EA, Speller AV, Walsh JL, Veselkov KA, Bunch J, Takats Z, Gilmore IS (2016). Investigation of the impact of desorption electrospray ionization sprayer geometry on its performance in imaging of biological tissue. Anal. Chem..

[13] Green FM, Salter TL, Gilmore IS, Stokes P, O'Connor G (2010). The effect of electrospray solvent composition on desorption electrospray ionisation (DESI) efficiency and spatial resolution. Analyst..

[14] Eberlin LS, Ferreira CR, Dill AL, Ifa DR, Cheng L, Cooks RG (2011). Nondestructive, histologically compatible tissue imaging by desorption electrospray ionization mass spectrometry. ChemBioChem..

[15] Green FM, Stokes P, Hopley C, Seah MP, Gilmore IS, O'Connor G (2009). Developing repeatable measurements for reliable analysis of molecules at surfaces using desorption electrospray ionization. Anal. Chem..

[16] Kertesz V, Van Berkel GJ (2008). Improved imaging resolution in desorption electrospray ionization mass spectrometry. Rapid Commun. Mass Spectrom..

[17] Campbell DI, Ferreira CR, Eberlin LS, Cooks RG (2012). Improved spatial resolution in the imaging of biological tissue using desorption electrospray ionization. Anal. Bioanal. Chem..

[18] Takats Z, Wiseman JM, Gologan B, Cooks RG (2004). Electrosonic spray ionization. A gentle technique for generating folded proteins and protein complexes in the gas phase and for studying ion–molecule reactions at atmospheric pressure. Anal. Chem..

[19] Venter A, Sojka PE, Cooks RG (2006). Droplet dynamics and ionization mechanisms in desorption electrospray ionization mass spectrometry. Anal. Chem..

[20] Wang LQ, May SW, Browner RF, Pollock SH (1996). Low-flow interface for liquid chromatography inductively coupled plasma mass spectrometry speciation using an oscillating capillary nebulizer. J. Anal. At. Spectrom..

[21] Pasilis SP, Kertesz V, Van Berkel GJ (2007). Surface scanning analysis of planar arrays of analytes with desorption electrospray ionization-mass spectrometry. Anal. Chem..

[22] Kertesz V, van Berkel GJ (2008). Scanning and surface alignment considerations in chemical imaging with desorption electrospray mass spectrometry. Anal. Chem..

[23] Van Berkel GJ, Ford MJ, Deibel MA (2005). Thin-layer chromatography and mass spectrometry coupled using desorption electrospray ionization. Anal. Chem..

[24] Swales JG, Tucker JW, Strittmatter N, Nilsson A, Cobice D, Clench MR, Mackay CL, Andren PE, Takats Z, Webborn PJ, Goodwin RJ (2014). Mass spectrometry imaging of cassette-dosed drugs for higher throughput pharmacokinetic and biodistribution analysis. Anal. Chem..

[25] Race AM, Styles IB, Bunch J (2012). Inclusive sharing of mass spectrometry imaging data requires a converter for all. J. Proteomics..

[26] Dill AL, Eberlin LS, Costa AB, Ifa DR, Cooks RG (2011). Data quality in tissue analysis using desorption electrospray ionization. Anal. Bioanal. Chem..

[27] Veselkov KA, Vingara LK, Masson P, Robinette SL, Want E, Li JV, Barton RH, Boursier-Neyret C, Walther B, Ebbels TM, Pelczer I, Holmes E, Lindon JC, Nicholson JK (2011). Optimized preprocessing of ultra-performance liquid chromatography/mass spectrometry urinary metabolic profiles for improved information recovery. Anal. Chem..

[28] Li, H.F., Jiang, T., Zhang, K.H.: Efficient and robust feature extraction by maximum margin criterion. Adv. Neural. Inf. Process. Syst. **16**, 97–104 (2004) 10.1109/TNN.2005.860852 16526484

