# Supplementary material for: Faster, More Reproducible DESI-MS for Biological Tissue Imaging
Source: J Am Soc Mass Spectrom. 2017 Jun 15;28(10):2090–8. doi: 10.1007/s13361-017-1714-z (PMC5594051; doi:10.1007/s13361-017-1714-z)
Supplement: Supplementary file 1 — (DOCX 2005 kb) [file 13361_2017_1714_MOESM1_ESM.docx]

Supplementary information

***Journal of the American Society for Mass Spectrometry***

**Faster, more reproducible DESI-MS for biological tissue imaging**

Jocelyn Tillner^a,d^, Vincen Wu^a^, Emrys A Jones^a,b^, Steven D Pringle^b^, Tamas Karancsi^c^, Andreas Dannhorn*^a^*, Kirill Veselkov*^a^*, James S McKenzie*^a^*, Zoltan Takats^a*^

*^a^ Computational and Systems Medicine, Department of Surgery and Cancer, Faculty of Medicine, Imperial College London, Sir Alexander Fleming Building, South Kensington, London SW7 2AZ, United Kingdom;*

*^b^ Waters Corporation, Altrincham Rd, Wilmslow, Cheshire East SK9 4AX, United Kingdom;*

*^c^ Waters Research Center, Záhony utca 7., C ép., 1. em., H-1031, Budapest,Hungary*

*^d^ NiCE-MSI, National Physical Laboratory (NPL), Hampton Road, Teddington, Middlesex, TW11* *0LW, United Kingdom;*

**Corresponding Author:**

*Zoltan Takats, [z.takats@imperial.ac.uk](mailto:z.takats@imperial.ac.uk)


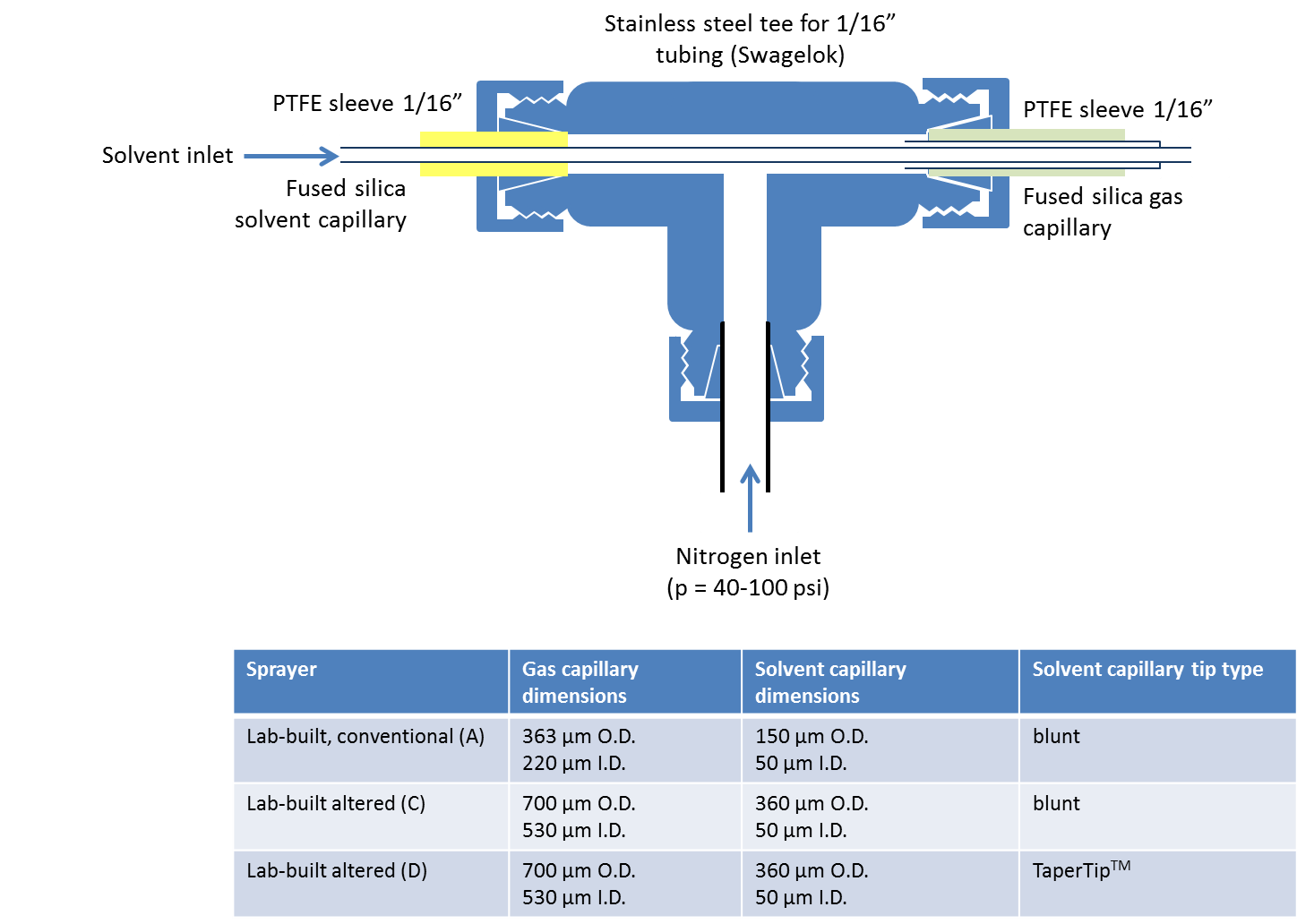


Supplementary Figure S 1: Schematic of the lab-built sprayer assembly and capillary dimensions for the various alterations.


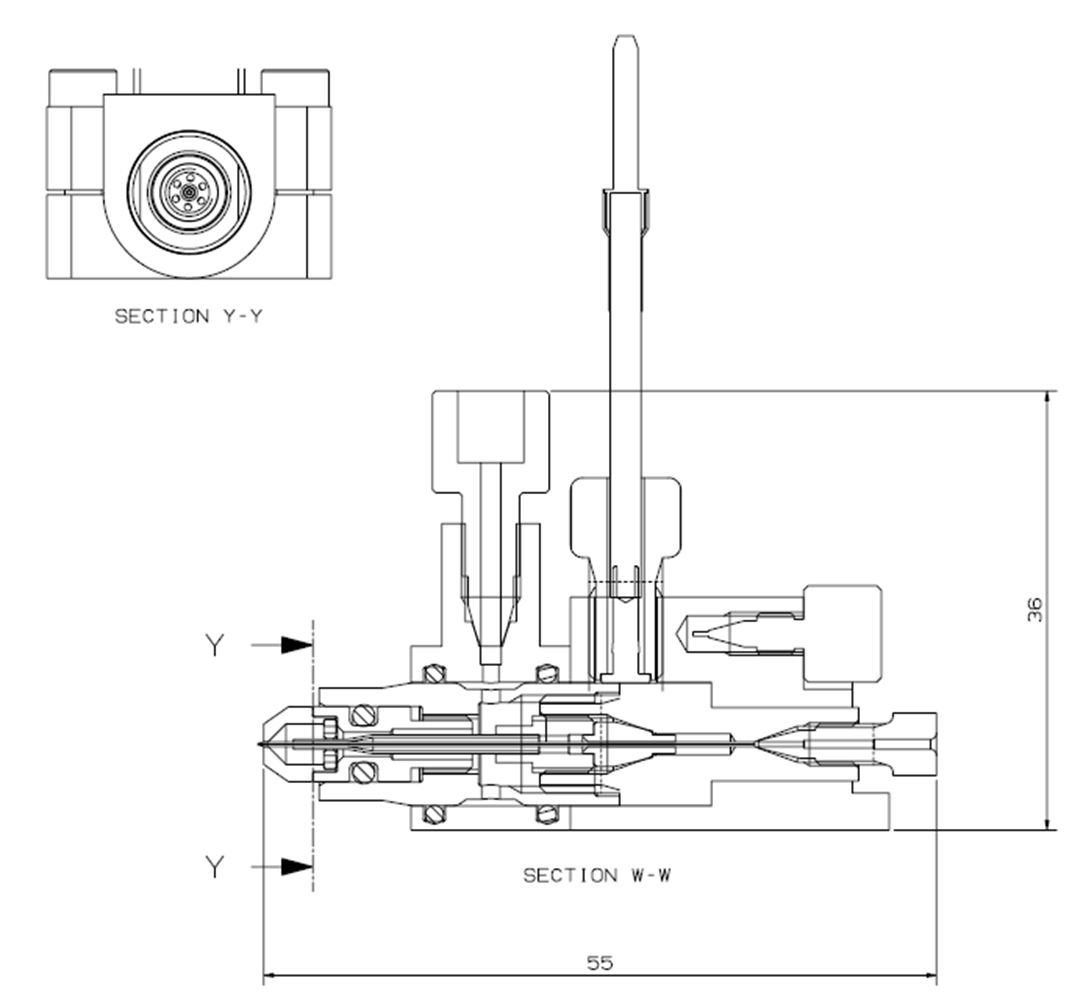


Supplementary Figure S 2: Schematic drawing for the novel sprayer constructed in collaboration with Waters Corporation.


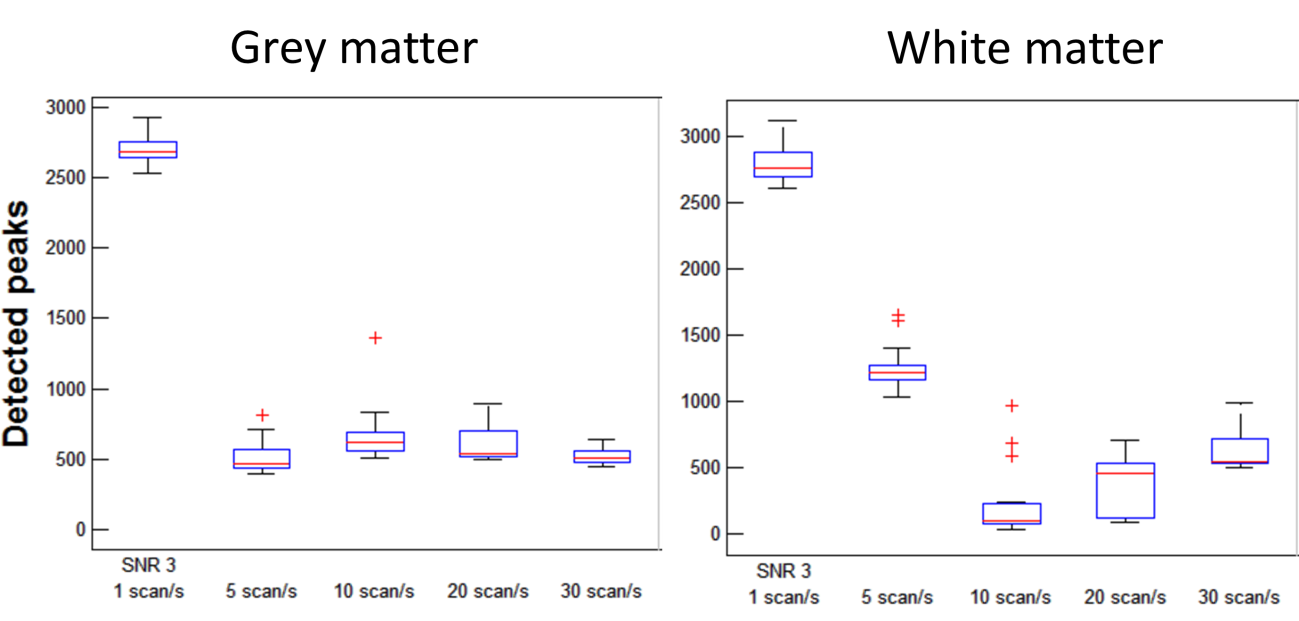


Supplementary Figure S 3: Number of spectral features at different scan speeds for 25 selected pixels in white and grey matter.


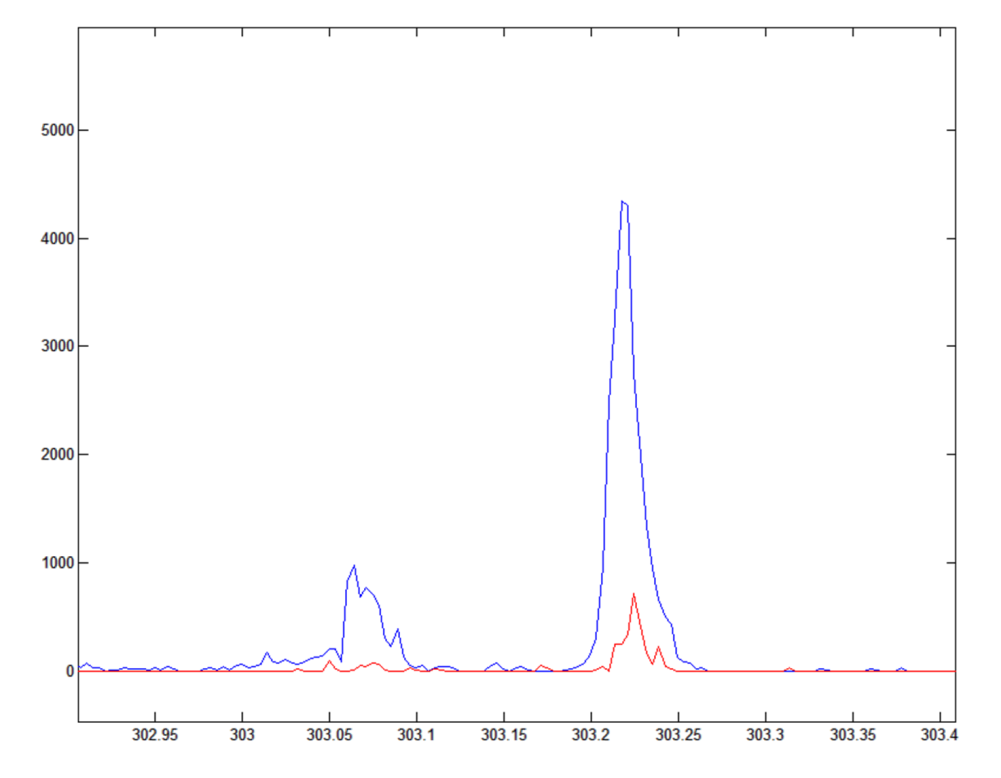


Supplementary Figure S 4. Illustration of peak shape deterioration for low intensity peaks. This leads to a nominal increase of the number of spectral features at high scan speeds. Blue plot shows a spectrum acquired at 1 scan/s, whilst the red line shows a spectrum recorded at 30 scan/s.


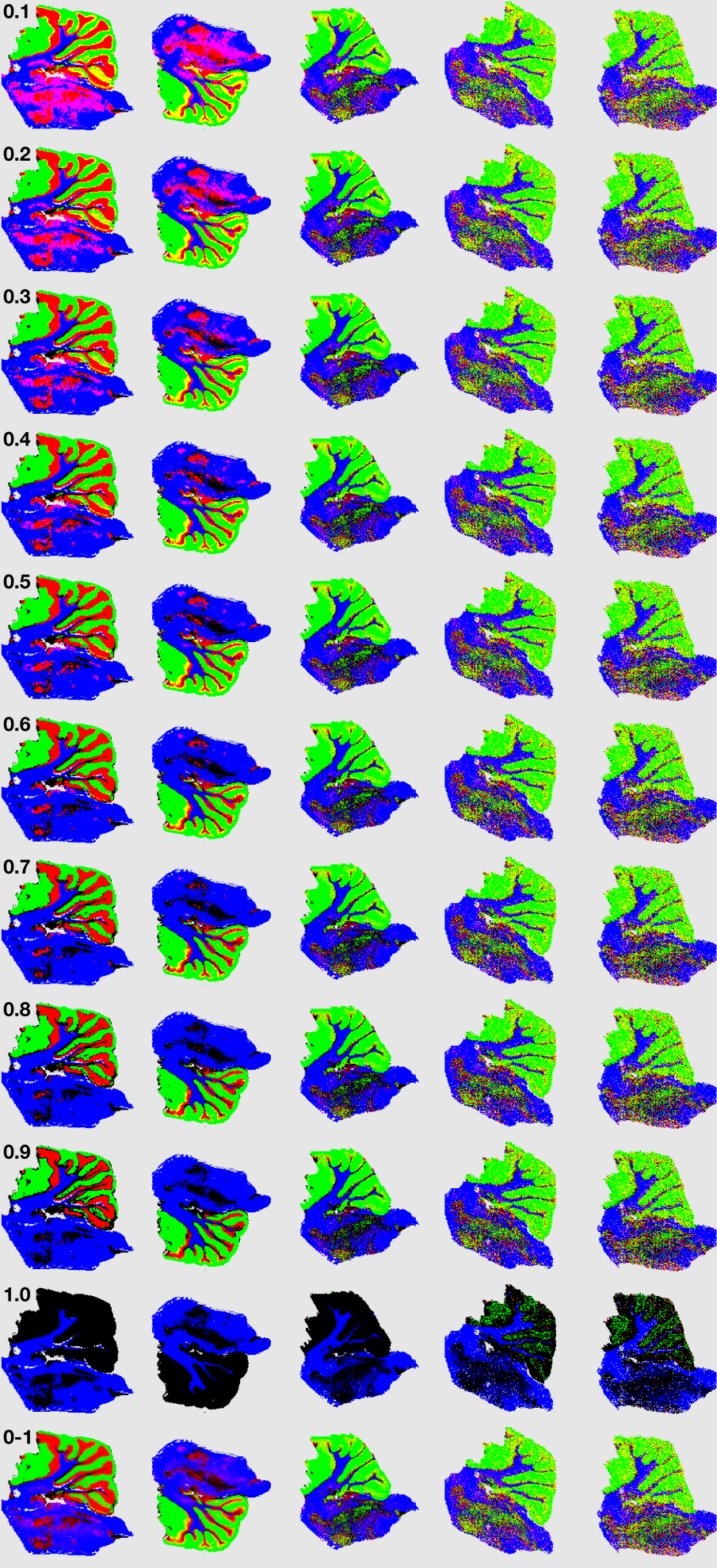


Supplementary Figure S 5. Tissue classification using different fixed probability thresholds (0.1 to 1) and a continuous probability scale (and colour gradients).


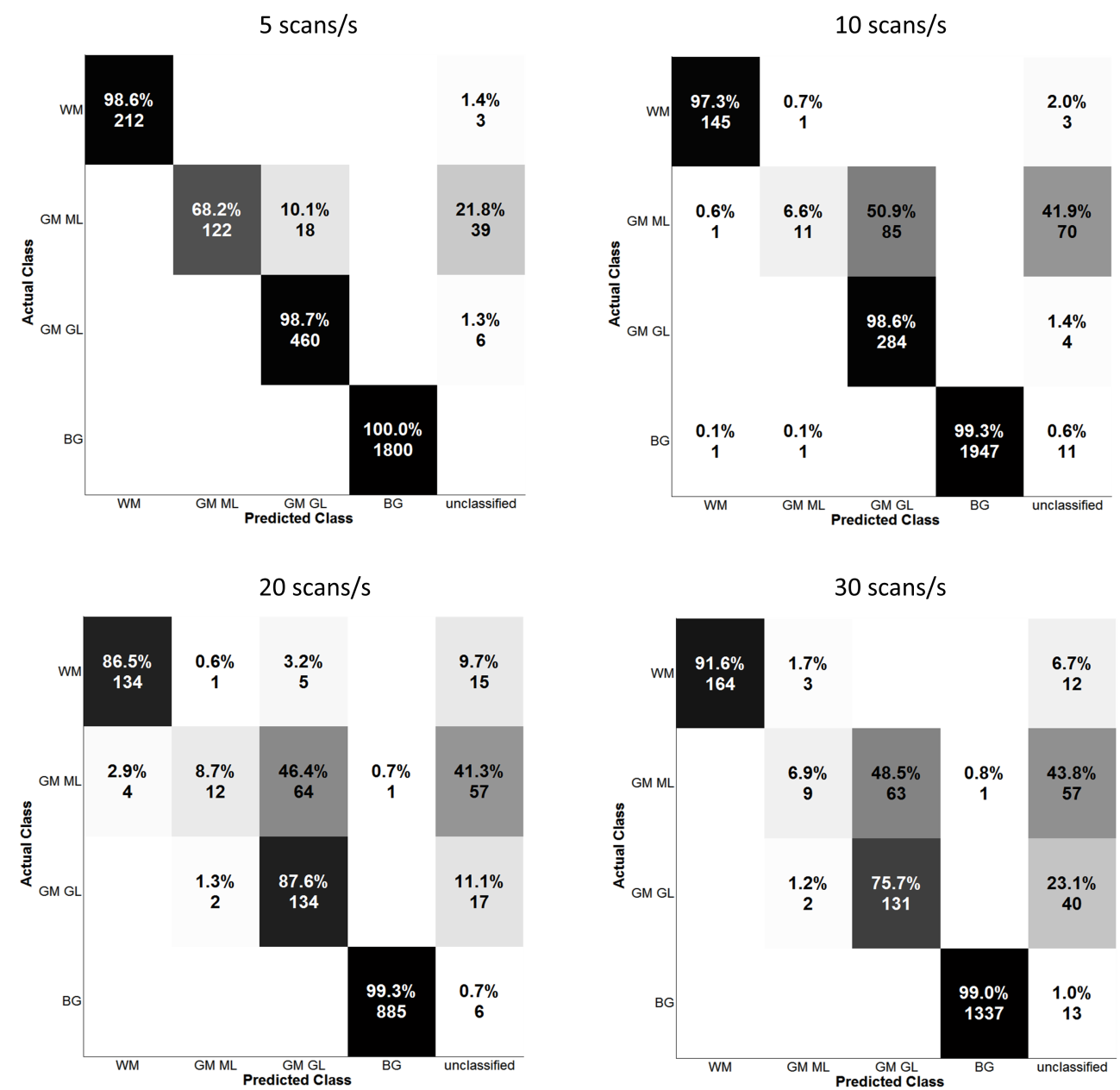


Supplementary Figure S 6: Confusion matrices for pixel classification of sections acquired at 5, 10, 20, and 30 scans per second and a prediction threshold of 0.9. A model was built using the data from the image acquired at 1 scan per second with pixels annotated by a histopathologist. All pixels in the other four images were classified using this model and a prediction threshold of 0.9. Predictions were validated against selected pixels annotated by the histopathologist. WM - white matter; GM ML - grey matter molecular layer; GM GL - grey matter granular layer; BG - background.

**Supplementary information regarding the TOF analyser and mass spectral resolution**

All DESI-QToF experiments were performed in sensitivity mode. Waters tune the transfer optics prior to the pusher to give two modes of operation; resolution and sensitivity. Resolution mode sacrifices approx. a factor of x3 in ions detected by reducing the energy spread of the ions entering the pusher using a ion optics and an entrance slit thus increasing the resolution, sensitivity mode allows nearly all of the ions (and a larger energy spread) to enter the pusher thus increasing intensity but decreasing resolution. For the experiments presented, mass spectral resolution was about 25 000 to 30 000.

When changing the mass spectrometer scan speed, the number of pushes decreases as the integration time decreases, therefore the measured intensity (number of ions collected per integration interval) decreases as the number of spectra (pixels) acquired per second increases.

ToF mass spectral resolution for a given species remains the same regardless of the integration time. The theoretical mass accuracy is a function of the ion count rate and resolution and is given by $\sigma\left( \mathrm{FWHM} \right)=\frac{{10}^{6}}{2.35R\sqrt{N}}$ where R is resolution @ FWHM (M/ΔM) and N is the number of ions counted (for the Waters detection system, N is not the intensity reported but is approx. I/30 (30 being the average ion area). Thus, increased resolution and increased intensity lead to better theoretical mass accuracy.

The spectral resolution of a mass analyser is both charge state and m/z dependent. Lower m/z and charge state ions have a lower resolution than higher charge state and m/z ions. Lower m/z ions have shorter flight times and so the jitter component due to the detection of the pusher pulse and detection electronics is a higher proportion of the flight time for lower m/z species than higher m/z species. Additionally the collisional cooling in the gas cell pre-pusher results in a smaller phase space injected into the pusher for high charge state ion than lower charge state ions.
